# Physicochemical Properties, Antioxidant Capacity, Prebiotic Activity and Anticancer Potential in Human Cells of Jackfruit (*Artocarpus heterophyllus*) Seed Flour

**DOI:** 10.3390/molecules26164854

**Published:** 2021-08-11

**Authors:** Ibna Suli Trejo Rodríguez, Luz Eugenia Alcántara Quintana, Paola Algara Suarez, Miguel Angel Ruiz Cabrera, Alicia Grajales Lagunes

**Affiliations:** 1 Faculty of Chemical Sciences, Autonomous University of San Luis Potosí, Universitaria, San Luis Potosí 78210, Mexico; ibna.trejo@uaslp.mx (I.S.T.R.); mruiz@uaslp.mx (M.A.R.C.); 2 Faculty of Nursing and Nutrition, Autonomous University of San Luis Potosí, San Luis Potosí 78240, Mexico; luz.alcantara@uaslp.mx (L.E.A.Q.); paola.algara@uaslp.mx (P.A.S.)

**Keywords:** jackfruit seeds flour, functional properties, antioxidant activity, dietary fiber, prebiotic, anticancer activity

## Abstract

The general aim of this study was to evaluate physicochemical properties, prebiotic activity and anticancer potential of jackfruit (*Artocarpus heterophyllus*) seed flour. The drying processes of jackfruit seeds were performed at 50, 60 and 70 °C in order to choose the optimal temperature for obtaining the flour based on drying time, polyphenol content and antioxidant capacity. The experimental values of the moisture ratio during jackfruit seed drying at different temperatures were obtained using Page’s equation to establish the drying time for the required moisture between 5 and 7% in the flour. The temperature of 60 °C was considered adequate for obtaining good flour and for performing its characterization. The chemical composition, total dietary fiber, functional properties and antioxidant capacity were then examined in the flour. The seed flour contains carbohydrates (73.87 g/100 g), dietary fiber (31 g/100 g), protein (14 g/100 g) and lipids (1 g/100 g). The lipid profile showed that the flour contained monounsaturated (4 g/100 g) and polyunsaturated (46 g/100 g) fatty acids. Sucrose, glucose, and fructose were found to be the predominant soluble sugars, and non-digestible oligosaccharides like 1-kestose were also found. The total polyphenol content was 2.42 mg of gallic acid/g of the sample; furthermore, the antioxidant capacity obtained by ferric reducing antioxidant power (FRAP) and 2,2-diphenyl-1-picrylhydrazyl (DPPH) was 901.45 µmol Trolox/100 g and 1607.87 µmol Trolox/100 g, respectively. The obtained flour exhibited good functional properties, such as water and oil absorption capacity, swelling power and emulsifier capacity. Additionally, this flour had a protective and preventive effect which is associated with the potential prebiotic activity in *Lactobacillus casei* and *Bifidobacterium longum*. These results demonstrate that jackfruit seed flour has good nutritional value and antioxidant and prebiotic activity, as well as potential protective effects and functional properties, making it an attractive food or ingredient in developing innovative functional products.

## 1. Introduction

The jackfruit (*Artocarpus heterophyllus*) is highly produced and consumed in the southeast of Asia and Brazil [1]. In Mexico, the production and consumption of jackfruit is recent and has increased in the last years because of its flavor and nutritional properties. The seeds however, have no use and are considered waste products, although they are rich in starch, proteins, crude fiber [2,3] and phytonutrients, such as lignans and isoflavones, which have wide-ranging health benefits [4]. In some countries to extend shelf life seeds are boiled or roasted are eaten as a snack [5]. Obtaining flour from these seeds has been considered another alternative for their use, but the conditions of the drying process for obtaining it are very variable [1,2,6,7,8]. The nutrients composition and some functional properties of seeds flour of jackfruit have been reported [1,2,8]. These studies, however, have only reported the global chemical composition of the jackfruit seeds flour, but the type of carbohydrates, lipids and total fiber have not been reported. Additionally, the antioxidant capacity in the flour has not been reported either. Regarding to biological activity as prebiotic effect and colon anti-cancer activity of jackfruits flour there are no report in the literature. The prebiotics component in jackfruits seeds has been reported, yet its effect has not been evaluated [9]. The prebiotic activity plays an important role in the health of intestinal microbiota which is associated with the prevention of the gastrointestinal diseases including colon cancer. Gastrointestinal diseases are a global health problem that has been increasing in recent years [10], and prebiotic consumption can active immune components from the gut lumen, and with intraepithelial lymphocytes, which modulate the innate immune barrier by secretion of IgA [11,12,13]. Additionally, it has also been demonstrated that prebiotic fermentation by anaerobic bacteria produces short-chain fatty acids (SCFAs), mainly acetic, propionic and butyric acids, whose increase antagonizes the growth of some pathogenic bacterial strains and favors mucin production in the colon [11]. For this reason, the objectives of this work were (1) to perform the drying kinetics of jackfruit seeds at different temperatures and to use a mathematical model to calculate the time required for obtaining a flour with a desired moisture content based on drying time, polyphenol content and antioxidant capacity, (2) to determine chemical composition and techno-functional properties and (3) in vitro evaluation of prebiotic effect and anti-cancer activity on human colon epithelial cell.

## 2. Materials and Methods

### 2.1. Jackfruit Seeds

The seeds were obtained in the Huasteca Potosina region (Huichihuayán, in the state of San Luis Potosí, Mexico) from 15 jackfruits. The weight of each seeds was obtained, and the seeds were then cleaned and stored in resealable bags at −20 °C until later use and analysis.

### 2.2. Drying Kinetics of Jackfruit Seeds

Because of the variability that exists in seed drying to obtain the flour, drying kinetics studies were performed to control the drying process. Seed samples were stored overnight at 4 °C before starting the experiments. At the start of each experiment, the seeds were cut into eight pieces, including the shell or aryl, with dimensions of 10 × 7 × 5 mm. About 50 g of these samples were spread in a square tray in a single layer. Drying experiments were performed using three replicates at the temperatures of 50, 60 and 70 °C in a forced air oven (model ULM 500, Memmert, Schwabach, Germany) with an air velocity of 1 m/s in all the experiments. The weight reduction of the samples during the drying process was recorded every 30 min with an electronic balance (±0.0001 g, Explorer model E12140, Ohaus, NJ, USA). Drying was stopped when consecutive weight measurements gave constant values. Average data are reported in drying curves. 

When the drying was complete, the samples were ground separately in a food processor (NutriBullet Rx) and then placed in Tyler sieves in order to obtain flour with a particle size of ≤250 µm. The flour was stored in airtight, amber bottles at room temperature until use.

The best temperature of drying was selected based on average drying time, polyphenol content and antioxidant capacity.

### 2.3. Mathematical Modeling of Drying Curves

Moisture content (*X*) was transformed to a dimensionless form referred to as the moisture ratio (X*) and was then related to the time for deriving the drying curves. The drying curves were then analyzed with Page’s model given by the following equation:

(1) $$X^{*}=\frac{X_{t}-X_{e}}{X_{0}-X_{e}}=exp^{{\left(-kt\right)}^{n}}$$

where *X (t)*, *X*_0_ and *Xe* are the moisture content at any time, at *t* = 0 and *t* = ∞ respectively; *k* is the drying constant (min^−1^), which is a phenomenological property representative of mass and heat transport phenomena; *t* is the drying time (min); and *n* is the dimensionless parameter of the Page equation. The goodness of fit was estimated by the coefficient of determination (*r*^2^). 

### 2.4. Analysis Performed in Jackfruit Seeds Flour

Polyphenols content and antioxidant activity tests were performed on flour obtained at the three temperatures with an average moisture content of 6.5 g/100 g. Chemical composition, techno-functional properties, prebiotic and anticancer activity assays were carried out only on the flour obtained at 60 °C because at this temperature, it was possible to achieve a short drying time without affecting the polyphenol content and antioxidant capacity.

### 2.5. Chemical Composition

Proximate analyses of moisture, proteins, lipids, ash, total carbohydrates and reducing sugars were performed based on established techniques [14]. The total dietary fibers (TDF) was determined using the enzymatic-gravimetric method [985.29 of the AOAC]. This methodology consists of the simulation of the digestion of the sample with the use of enzymes. One gram of defatted flour was gelatinized with α-amylase (at 95 °C, pH 6, for 15 min) and afterward was digested with protease at 60 °C, pH 7.5 for 30 min. Finally, the sample was incubated with amyloglucosidase (at 60 °C, pH 4.5, for 30 min). After the enzymatic treatment, the sample was filtered and simultaneously washed with 95% ethanol and acetone. The recovered filtrate was dried, and the protein and ash content were determined for calculating the TDF content. The TDF was determined in quadruplicate.

### 2.6. FTIR Infrared Spectroscopy

In order to determine the main functional groups in the flour, a Nicolet iS 10 FTIR spectrometer (Thermo Scientific, Madison, WI, USA) equipped with a universal attenuated total reflection (ATR) detector was used. The spectrum of the sample and its duplicate were recorded in transmittance mode with a 4 cm^−1^ resolution in the region of 4000–300 cm^−1^ at room temperature [15].

### 2.7. Carbohydrates Characterization by HPLC

Carbohydrates characterization was performed using the methodology reported by [16]. 20 μL of sample were analyzed using an 1100 Series HPLC chromatography system (Agilent Technologies, Santa Clara, CA, USA). The equipment includes a degassing device, a quaternary pump and a refractive index detector (Waters 410, Waters Corporation, Milford, MA, USA). An ion exchange column was used as a stationary phase (model HPX-87C, 7.8 mm internal diameter × 300 mm, Aminex, Hercules, CA, USA). The mobile phase was deionized HPLC grade water with a flow rate of 0.5 mL/min at 75 °C. The program Empower QuickStart 5.0 was used as a control and data analysis system. Arabinose, fructose, galactose, glucose, lactose, maltose, mannose, ribose, sucrose and xylose, as well as chicory inulin, all with purities greater than 98% and from Sigma-Aldrich (St. Louis, MO, USA) were used as standards. The resolution time was 20 min, and carbohydrates were identified by comparing the retention times with the corresponding references standards. Quantification was obtained according to the reference carbohydrate calibration curve (*r* = 0.99).

### 2.8. Lipids Characterization

The lipids were extracted using the Soxhlet extraction method with analytical grade ether as the reflux solvent for 4 h. At the end of the extraction, the oil was recovered from the mixture by distillation on a rotary evaporator. Sodium sulfate was added to remove moisture from the extract which was stored at 4 °C until use.

### 2.9. Sample Derivatization and Fatty Acid Profiles

Fatty acid methyl esters (FAME) were prepared according to the AOAC 969.33 method. A methanol solution was prepared with NaOH (0.1 g) in methanol (5 mL), and the mixture was heated to dissolve the sodium hydroxide. The extract was placed in a test tube (900 mg) after, 0.8 mL of the methanol solution was added and the mixture subjected to a microwave cycle method using a Discover System Model 908005 (CEM, Watertown, MA, USA) with the following conditions: 100 °C, 150 W, 290 Psi, with a running time of 3 min and a warm-up time of 10 min, with high agitation. Subsequently, 1 mL of boron trifluoride in methanol was added, and a microwave cycle was applied to the same conditions. Finally, 1 mL of trimethylpentane was added, and again a microwave cycle was applied. The sample obtained had two phases, and the organic phase was used for the injection into the chromatograph.

The FAME were analyzed on a 6890N gas chromatograph of an Agilent Technologies model 5973 system, equipped with a ZB-WAX column (30 m in length, 320 µm internal diameter and 0.50 µm of thickness) and an FID detector. The carrier gas was helium. The temperature of the column was initially 100 °C (for 5 min) and was gradually increased to 240 °C for 4 °C/min. This temperature was maintained for 20 min. The injector temperature was 240 °C, and 1 μL of the organic phase was injected twice. The identification was performed by comparing the retention times of the FAME standards, which consist of a mixture of 37 fatty acid methyl ester.

### 2.10. Total Polyphenols Content and Antioxidant Capacity

The total polyphenol content was evaluated in the flour obtained from the seeds. For extraction, the sample was allowed to stand for 24 h in the methanol (80%) at 4 °C. The solution was filtered for evaluating the total phenolic content following the Folin-Ciocalteau reagent assay [17].

The antioxidant capacity was measured using two methods. The first was 2,2-diphenyl-1-picrylhydrazyl (DPPH) radical assay following the protocol from Santos-Zea [18], and the results were expressed in µmoles of Trolox/100 g. The second was the ferric reducing antioxidant power (FRAP) assay following the method [17] and the results were expressed as μmoles of Trolox/100 g.

### 2.11. Techno-Functional Properties

#### 2.11.1. Water and Oil Absorption Capacity

One g of sample was dispersed in 10 mL of deionized water or in 6 mL of soybean oil in a centrifuge tube. After stirring, the sample for water and oil absorption was centrifuged at 4280× *g* for 60 min and 3077× *g* for 25 min, respectively, the measurements were performed at room temperature (25 °C). The supernatant was decanted, and the water and oil absorption were calculated as a percentage [19].

#### 2.11.2. Emulsifying Capacity

One g of flour was suspended in 20 mL of deionized water, and then 7 mL of soybean oil was added to it. The mixture was emulsified with a T 25 digital ULTRA-TURRAX (IKA, Wilmington, NC, USA) at 192× *g* for 1 min. The emulsion obtained was centrifuged at 1731× *g* for 60 min. the measurements were performed at room temperature (25 °C). The emulsifying capacity was calculated as a percentage of the height of emulsified layer/the height of the whole layer in the tube) × 100 [20].

#### 2.11.3. Swelling Power and Water Solubility Capacity

The 500 mg of flour (*W*_1_) were placed in a tube with 20 mL of distilled water and weighed (*W*_2_). The sample was heated in a water bath at 40, 50, 60 and 70 °C for 30 min, then a centrifugation was performed at 2292× *g* for 10 min. The supernatant was separated and used to determine the solubility, and the tube and its contents were weighed (*W*_3_). For solubility, 10 mL of each supernatant (*Vs*) were taken and placed in a crucible at constant weight (*W*_4_). The crucible with the sample was dried in a forced air oven at 105 °C for 12 h [8]. After constant weight (*W*_5_) of the sample, these properties were calculated with the following equations:

(2) $$\mathrm{Swelling}\;\mathrm{power}\;\left(g/g\right)=\frac{W_{3}-W_{2}}{W_{1}}$$

(3) $$\mathrm{Solubility}\;\left(\%\right)=\frac{W_{5}-W_{4}}{Vs}\;\times \;\;\frac{100}{W_{1}}$$

#### 2.11.4. Viscosity Evaluation

To evaluate the viscosity of the flour, a RheoPlus/32 rheometer (Anton Paar, Ostfildern-Scharnhausen, Germany) was used, which is equipped with a parallel stainless steel plates (θ = 60 mm) geometry. The temperature was increased from 30 to 80 °C. A gap of 0.5 mm between the plates was used. The concentrations evaluated were 1, 3 and 5 g/100 mL, and the amount of sample used was 1.5 mL. The viscosity Pa·s was determined as a function of the increase in the shear rate (γ) from 10 to 600 s^−1^.

### 2.12. Prebiotic Extracted from Jackfruit Seed

The prebiotic extraction was carried out according to a published procedure [9] with some modifications. Fresh jackfruit seeds were cut and mixed with 80% ethanol and then ground in a processor to obtain smaller particles. This mixture was allowed to stand 24 h at 4 °C. The sample was then filtered and centrifuged at 1000 rpm/1 h at 4 °C. The supernatant was concentrated on a heating plate at 60 °C with shaking until the content was reduced to half. The concentrate was dehydrated in a silicone tray at 60 °C for 24 h in a forced air oven.

### 2.13. Prebiotic Effect of Jackfruits Seed Flour and Jackfruits Seeds Extracted

The prebiotic effect of flour and prebiotic extracted was performed using the methodology [21] with some modifications. The growth of *Lactobacillus casei* and *Bifidubacterium longum* was evaluated using a MRS culture medium (Difco, San José, CA, USA) with the same composition but replacing the carbohydrates source. Different sources of carbohydrates such as *Agave salmiana* fructans, commercial inulin and whole wheat flour were used because they are already recognized as prebiotics and we consider it important to compare them with the prebiotic effect of jackfruit flour. The growth of these bacterial was also evaluated in commercial MRS media and MRS without carbohydrates source and were used as control. All carbohydrates source including jackfruit flour were evaluated to 20 g/L of concentration. The broth MRS for *B. longum* were supplemented with 0.05 g/L of L-cysteine. *L. casei* was inoculated was 0.06% (*v*/*v*) corresponding to approximately 1 × 10^8^ CFU/mL in all culture media (25 mL) and incubated at 37 °C/24 h. The concentration of each inoculum was verified through plate count with MRS agar using the Miles and Misra methods [22] the bacteria were incubated anaerobically at 37 °C for 48 h and the growth was expressed as CFU/mL.

### 2.14. Anticancer Activity in Human Colon Epithelial Cells

#### 2.14.1. Cell Cultures

The human colon epithelial cell lines (normal) CRL1831, (cancerous, grade 1–2) HT29 and (cancerous, grade 3–4) SW480 were transferred to 25-cm^2^ culture flasks and grown in Dulbecco’s modified Eagle medium (DMEM, Gibco, Invitrogen Corp., Carlsbad, CA, USA) supplemented with 10 % of fetal bovine serum (FBS) (Gibco) and antibiotics (100 U/mL penicillin, 100 µg/mL streptomycin, 0.25 µg/mL amphotericin). The cells were cultured in a humidified incubator with 5 % CO_2_ and 95 % air atmosphere at 37 °C. When the cells grew to 80 to 90 % confluence, they were washed with PBS and were trypsinized with 0.5% trypsin-EDTA (Gibco). In addition, the cells were used in subsequent experiments.

#### 2.14.2. Treatments

The HT29, and SW480 cells were seeded in 96-well plates at a density of 1 × 10^5^/well with PBS. The PBS was replaced with DMEM supplemented only for the control for the treatments, PBS was replaced with DMEM-supplemented with jackfruit flour at a concentration of 50 µg/mL, 100 µg/mL, 500 µg/mL and 1 mg/mL. The cells were incubated in a humidified incubator with 5% CO_2_ at 37 °C for 24 h.

#### 2.14.3. Cytotoxicity Assay

Viability was assessed with a thiazolyl blue tetrazolium bromide (MTT) assay at 5 mg/mL, at 24, 48 and 72 h, measuring absorbance with a spectrophotometer at a wavelength of 570 nm.

#### 2.14.4. Antioxidant Assay

The CRL1831, HT-29, SW480 colon epithelial cells stored in PBS (100 µL) were homogenized adding proteinase k (2 µg/µL, Sigma-Aldrich, placed in a water bath for 24 h at 25 °C. After this period, proteinase k was inactivated, the samples were centrifuged at 3000× *g* at 4 °C for 5 min. The cell-free supernatants from all the treatments and controls were collected in new sterile tubes. The activities of superoxide dismutase (SOD) and catalase (CAT) enzymes were measured using a colorimetric assay kit according to the manufacturer’s protocol: SOD (EC 1.15.11) Assay kit item no. 19160, CAT (EC 1.11.1.6) Assay kit item no. CAT100 (Sigma-Aldrich), using a plate reader.

### 2.15. Statistical Analysis

All analyses were carried out in triplicate, and the data are given as the mean ± standard deviation. An analysis of variance (ANOVA) and Fisher’ least significant difference (LSD) test were performed at a level of *p* < 0.05 and *p* < 0.01 for all response and colon cell respectively using the GraphPad Prism 9.1 software (GraphPad, San Diego, CA, USA).

## 3. Results and Discussion

### 3.1. Jackfruit Seeds

The individual weight of each seed was between 6 and 8 g, and these seeds represent 8.5% of the total weight (0.56 ± 0.3 kg) of jackfruit, which is an attractive percentage for use in flour manufacturing. This value was similar to that reported by [23] who indicated that the percentage of seed weight in jackfruit is between 8 and 15%.

### 3.2. Drying Kinetics of Seeds

**Figure 1:**
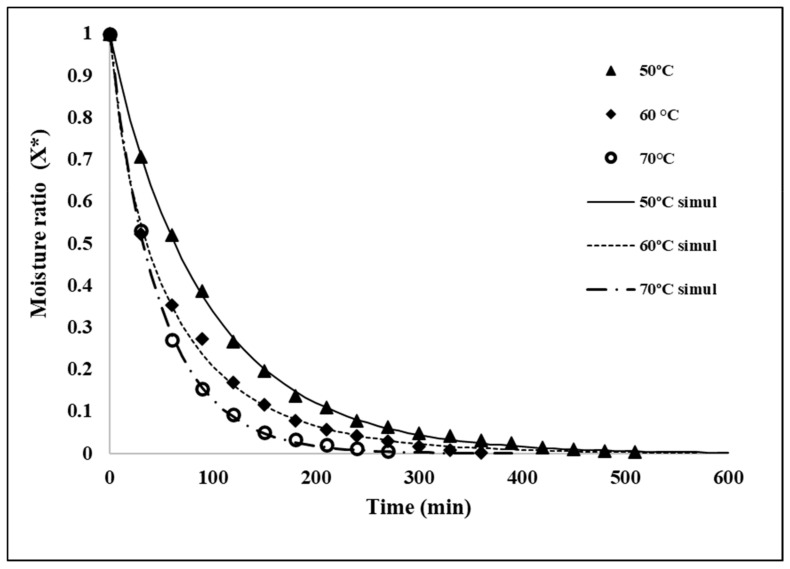
Experimental evolution and the predicted moisture ratio of jackfruit seeds’ flour with drying times at 50, 60 and 70 °C.

The experimental values of the moisture ratio during the drying of jackfruit seeds at different temperatures are shown in Figure 1. The shape of these curves is typical for food products with a drying process that is controlled by internal water transfer. Significant differences (*p* < 0.05) were observed between samples dried at 50, 60 and 70 °C to reach the equilibrium moisture. In Figure 1, the curves predicted with Page’s equation are also shown. The values of r^2^, n and k (min^−1^) calculated with Page’s equation are given in Table 1. A good correlation between experimental and predicted values of the moisture ratio with r^2^ values higher than 0.99 was observed. Therefore, Page’s equation is a good model for describing the drying of jackfruit seeds and can be used to calculate the required or desired moisture content of samples at 50, 60 and 70 °C. These results suggest that using any of these temperatures is not required long term to obtain a stable product with a moisture content of less than 10% of the flour. For example, from this figure the time required to obtain a flour with 6.5% of moisture at 60 °C was 8.5 h.

### 3.3. Chemical Composition

The chemical composition values of flour are presented in Table 2. The mean values of moisture and total ash content were of 6.54 g/100 g and 3.25 g/100 g, respectively. With this moisture content, the flour was stable for 12 months at 25 °C and a relative humidity of 50%. This confirms that a moisture content of less than 10% is required for stable powdered products. With respect to the other biomolecules, the carbohydrates were the major highest component of jackfruit seeds’ flour (73.8 ± 1.06 g/100 g), which coincides with other studies reported [1,2,7]. The values of this study, however, were lower than those reported by Ocloo et al. [2] (79.3 g/100 g). The protein content of the flour was 14 ± 0.15 g/100 g; this value is similar to [2,7]) but [1] reported higher values. According to those reported by Lima et al. [1], the protein content of this flour is usually higher than that obtained from wheat flour with an approximate protein content of 10 g/100 g. This value qualifies jackfruit flour as a protein-rich product, and it could be used as an ingredient to prevent malnutrition. The lipids content was the lowest in the flour; the mean value was 1.3 g/100 g and is in the range of [2,8]. The differences observed between the values of chemical composition reported in the literature and those of this work can be attributed to the drying process and jackfruit variability. 

A high content of total dietary fiber (TDF) was found in the flour (31.5 g/100 g), which suggests that this flour is an ingredient with a high fiber content. The TDF of this flour has not been reported by other authors; only crude fiber was reported (3.19 and 3.72 g/100 g) by [2,20], respectively. The TDF value is greater than for whole wheat flour that has a value ranging from 11 to 14 g/100 g or for flours obtained from quinoa, oats and corn that have values of 4 to 5 g/100 g in fiber [24]. 

### 3.4. FTIR Analysis

**Figure 2:**
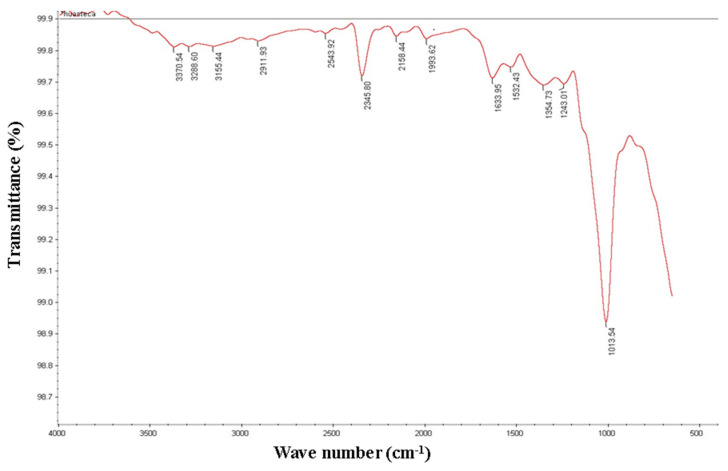
Jackfruit seeds flour spectra obtained using FTIR-ATR spectroscopy.

Figure 2 shows the FTIR spectra signal and the chemical bonds identified in jackfruit seed flour. The infrared analysis confirmed the presence of carbohydrates and proteins in flour samples made from jackfruit seeds. A typical peak appears in the region of 1013 cm^−1^, which reflects the presence of carbohydrates. This value is in the range of 1189–953 cm^−1^ reported by [1], and this band is the result of vibrations, stretching and deformation of bonds C-C, C-O and C-H, respectively [25]. The value of 1633 cm^−1^ and 1533 cm^−1^ have been related to amide group characteristics of proteins [1]. The range of 2500–2000 cm^−1^ the bands correspond to double and/or triple bonds and aromatic amino acids [26], which suggests that these types of component are present in the flour. The spectral region between 3000 and 3600 cm^−1^ corresponds to OH stretching vibration, which has been associated with moisture content [13]. No signals were identified in this region because the moisture content of the flour was below 10%.

### 3.5. Carbohydrates Characterization by HPLC

According to the HPLC analysis, five types of sugars were identified and quantified in jackfruit seeds flour (Table 3). The main carbohydrates identified were mono- and disaccharides like sucrose (35 mg/g), glucose (29 mg/g) and fructose (23 mg/g). According to the standard used, 20 mg/g corresponds to carbohydrates with a polymerization degree greater than 5, which according to the literature [27] could correspond to fructans, oligosaccharides, inulin, resistant starch, cellulose, hemicellulose, gums and pectin. Thus, it will be interesting to identify these compounds using the corresponding standard. In a lower content, 1-kestose (4 mg/g) was identified, which has been described as a non-digestible oligosaccharide considered a prebiotic because of its structure [28]. These types of compounds are relevant because they are not absorbed into the gastrointestinal tract and are not hydrolyzed by human enzymes and thus act as a substrate for endogenous bacteria of the intestine [28].

### 3.6. Lipids Characterization

Table 4 shows the percentages obtained for each fatty acid identified. The average of saturated fatty acids (SFAs) was 49.13 g/100 g, and the main components of SFAs were palmitic acid, tricosanoic acid and stearic acid. With respect to monounsaturated fatty acids (MUFAs), only oleic acid was identified with a total percentage of 4.15 g/100 g. The average percentage of polyunsaturated fatty acids (PUFAs) was 46.72 g/100 g. Linoleic acid, which is an omega-6 fatty acid, was the most dominant, with a relative percentage of 35.11 g/100 g. Furthermore, linolenic acid, arachidonic acid, eicosapentaenoic acid (EPA) and docosadienoic acid were also found. The total of monounsaturated and polyunsaturated fatty acids was 50.87 g/100 g, which is slightly higher than the SFAs. Some of these fatty acids were previously reported in jackfruit seeds [29], others SFAs such as dodecanoic, tridecanoic, tetradecanoic, pentadecanoic and heptadecanoic were also reported by these authors. However, the PUFAs such as arachidonic, eicosapentaeoic and docosadienoic were not reported by these authors. The high content of these fatty acids is relevant because they participate in the control and prevention of cardiovascular diseases, in the control and prevention of rheumatoid arthritis and in the metabolism of HDL lipoproteins [30]. These results suggest that for this flour the unsaturated/saturated fatty acids ratio was 1:1, which could be considered a good balance between both fatty acid type. The relation of ω6/ω3 was 5:1, although some studies report that the ideal ratio in a diet of ω6/ω3 to prevent cardiovascular risk is 1:1 or 1:2 [31]. In contrast, other authors suggest that for the secondary prevention of cardiovascular diseases a ratio of 4:1 has been associated with a 70% reduction in total mortality [32]. German-Austrian-Swiss recommendations stated that ω6 and ω3 fatty acids together should contribute 7 to 10% of the total energy intake with a ratio of linoleic acid (ω6) to α-linolenic acid (ω3) of 5:1 [33]. Additionally, this ratio was also similar to the value obtained for maize pollen (ZP 5557 Lady Fingers, (Maize Research Institute in Zemun Polje. Belgrade, Serbia) which was considered a good balance between ω6 and ω3 fatty acids [34].

### 3.7. Total Polyphenol Content and Antioxidant Capacity

The total polyphenol content (TPC) and antioxidant activity were determined in the three flours obtained at different temperatures. In Table 5, these results are presented, and the temperature used for the production of flour showed no significant difference (*p* > 0.05) in polyphenol content. It was observed, however, that the polyphenol content decreases when the temperature increases. For this reason and for the process time, we considered 60 °C as the average temperature adequate for obtaining the flour. Information on polyphenol content in flour obtained from jackfruit seeds is scarce. The TPC has only been reported in jackfruit seeds (2.12 ± 0.009 µg gallic acid/mg extract) [35], which is similar to the value reported in this work. [29], however, reported values between 1.31 and 213.41 mg/kg, which were dependent on the solvent and extraction method. The solvent and extraction method used for the isolation of antioxidants is an important factor that can affect the polyphenol content value. The polyphenol profile of jackfruit seeds was also reported by [29], where 12 types of polyphenols were found. Furthermore, 5-caffeoylquinic acid was the main component, and the presence of carotenoids was also indicated. Additionally, [4] also showed that jackfruits seeds contain polyphenols such as lignans and flavones. These compounds have benefits in the uptake of free radicals, protect different cell organelles, can prevent the oxidation of various cellular compounds and protect the human body against damage by reactive oxygen species [17] therefore, they could then provide beneficial effects in the prevention of coronary heart disease.

The antioxidant capacity of ingredients derived from vegetables has been a relevant field in the last years. The antioxidant capacity was determined by DPPH radical and FRAP. DPPH radical is a compound that is capable of generating free radicals and has been widely used to evaluate the ability to capture free radicals in compounds with antioxidant activity. The values of DPPH radical ranged from 1579 to 1617 µmol Trolox/100 g are shown in Table 5. These results revealed an increasing trend in radical scavenging activity with an increasing drying temperature. This high antioxidant capacity can be explained because a high temperature may lead to the formation of new compounds with higher antioxidant capacity [36]. These authors indicated that the increase in antioxidant capacity in plums dried at 60 and 85 °C was the result of an increase in hydroxymethylfurfural, which is an intermediate in Maillard reaction products (MRPs) production. The increase in DPPH radical value was also reported in hot air drying of pumpkin flour [37] and cowpea seed thermally processed [38]. The DPPH radical values of this study were higher than okra flour (0.309 mM Trolox/100 g) [39] and that in some drinks such as tea (631 µmol Trolox/100 mL) and orange juice (249 µmol Trolox/100 mL) but lower than the fiber-rich fraction of chia (488 µmol Trolox/g) [40]. Higher phenolic compounds and DPPH radical values were obtained in this study in comparisons to rye flour (0.08 to 0.58 mg gallic Acid/g d.m. and 235 µg Trolox/100 g d.m, respectively) [41].

The FRAP is a method used to evaluate the ferric reducing capacity by electron transfer. Antioxidant compounds cause the reduction of the ferric (Fe^3+^) to the ferrous (Fe^2+^) because of their reductive power [42]. The FRAP values are shown in Table 5 and were significantly (*p* < 0.05) reduced in the flours obtained at 60 and 70 °C, which is correlated with the trend observed in the polyphenol content. The FRAP and DPPH radical scavenging activity assays, however, determine antioxidant capacities through a different mechanism; therefore, these cannot be correlated [38]. It has been reported that thermal processing reduces TPC and FRAP and increases the DPPH value [38]. The antioxidant power of this flour is a relevant fact since it could be used to counteract reactive oxygen species, which trigger a cascade of diseases associated with oxidative stress that can damage human health.

### 3.8. Techno-Functional Properties of Flour

The techno-functional properties are important in food systems for the development or implementation of new products.

#### 3.8.1. Water Absorption Capacity (WAC)

The WAC is referred to as the ability of a material to retain water, such as linked, hydrodynamic and physically trapped water, under centrifugation conditions [40]. The WAC value of the flour was 463 mL/100 g ± 1.06. This result suggests that the flour has a good WAC, which can be associated with the increase of porosity, as well as the exposition of amino acid residues because of protein denaturation, starch gelatinization and raw fiber swelling during drying [20,43]. Fiber structure and the high proportions of hemicellulose and lignin may also augment WAC [40]. WAC is an important parameter in food processing; for example, a high WAC helps to preserve the freshness of bread, cakes and sausage and can be used as a soup thickener [2,8,19]. However, reported lower values than this study, this difference could be due to process conditions for obtaining the flour or to the effect that in our study the whole seed was used, and in the two studies the thin brown spermoderm was removed.

#### 3.8.2. Oil Absorption Capacity (OAC)

A good oil absorption capacity (OAC) of 34.83 ± 0.001 g/100 mL was obtained in this flour, which is higher than that reported for chia flour [40]. The high OAC is the result of protein denaturation during drying because it has been reported that protein hydrophobicity plays an important role in oil absorption [17], and can also depend on surface characteristics, total charge density and the hydrophobicity of fiber particles [42,44]. Additionally, it was reported that dietary fiber has the ability to retain fat and has been related to the capacity to decrease serum cholesterol levels and remove excess fat from the human body [42,45]. This property is important because fat can improve the flavor of foods and then, this flour could be a high fat and flavor retainer and may therefore find useful application in food systems such as ground meat formulations [2].

#### 3.8.3. Water Solubility Capacity (WSC) and Swelling Power

With respect to the water solubility capacity (WSC) and swelling power, Table 6 shows the results at different evaluated temperatures. Low WSC values were obtained and were directly correlated with increasing temperature. The range of WSC was 0.087 to 0.15%, and no significant differences (*p* = 0.069) were observed between the evaluated temperatures. A low solubility value may be related to only a slight degradation of starch and leads to having few soluble molecules in the flour [46]. This behavior was similar to that reported in earlier studies when the solubility and swelling power of jackfruits seed starch were evaluated [5]. These authors reported a significant increase above 75 °C; probably because of this, higher temperatures are required for improving these properties in the flour. Our results, however, were lower than reported by [7,8]. 

The swelling power was also directly proportional to the temperature used, and no significant difference (*p* = 0.112) were observed, with the values ranging from 4.06 to 5.15%. The increase of swelling power caused by temperature can be explained because more water is retained when the temperature increases, and then the flour begins to swell and volume augments [20]. Similar results were reported by these last authors when they used boiled jackfruit seeds meal. WSC and swelling power are the evidence of an interaction between the amorphous and crystalline areas, and they are influenced by amylose and amylopectin characteristics [47]. It was reported that WSC and swelling power is related with amylose content, slow amylose content increases these properties in starches soft wheat and bean [48]. Swelling power of this flour tends to increase in contrast to its solubility; this same behavior has been reported for tropical tuber flour [47].

The emulsifying capacity (EC) is a molecule’s ability to act as an agent that facilitates the solubilization or dispersion of two immiscible liquids [40] and it is also an important property for the food and pharmaceutical industry. The EC of jackfruit seeds’ flour was 45.27 ± 2.79 g/100 g, which was higher than that of okra flour [39] but lower than that of chia flour [40]. The higher EC of chia flour may be caused by the protein concentration, which is higher than that in the jackfruit seeds flour. In addition, it has been reported that this property may be influenced by protein hydrophobicity, pH, solubility and proteins concentration [39]. It is important to mention that the swelling value, emulsifying capacity and fat absorption of flour defines its use and application in food systems or complex matrices because this can predict whether it will confer flavor, texture or any other property to food [49].

#### 3.8.4. Dynamic Viscosity Evaluation

**Figure 3:**
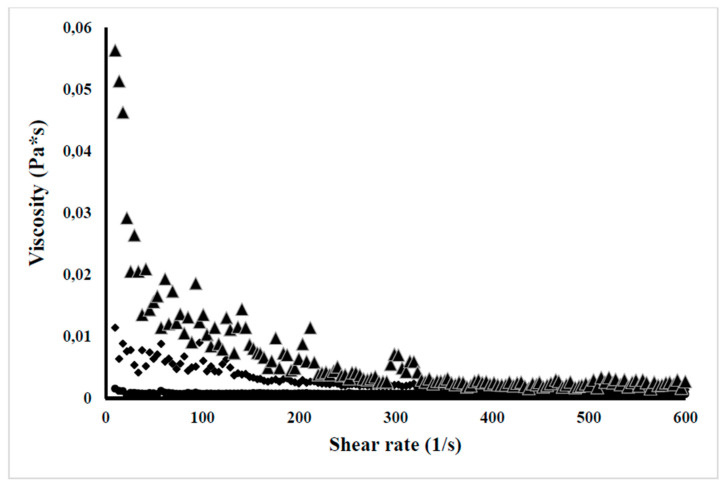
Viscosity dispersions of jackfruit seeds’ flour versus the shear rate at different concentrations (●1, ♦3, ▲5 g/100 mL).

The dynamic viscosity results of the three flour concentrations are shown in Figure 3. Dynamic viscosity was dependent on the shear rate, which is a typical characteristic of a non-Newtonian fluid, because the flour developed a lower viscosity when the cutting speed increased. It was observed that there is a gradual increase of the viscosity when the flour concentration increases. Significant differences were observed between the three concentrations evaluated (*p* < 0.05), the viscosity value to 5% was 0.056 ± 0.0002 Pa·s, while for the concentrations of 1% and 3% the maximum viscosity values reached were 0.0013 ± 0.00001 and 0.011 ± 0.00025 Pa·s, respectively. The increases of viscosity values in dispersions are associated with the higher fiber content, and some components of fiber, such as β-glucans, pectins, hemicelluloses, and cellulose, can retain more water to form a high viscosity suspensions [50]. The other components of flour like proteins, starch and lipids also play an important role in the final viscosity as was demonstrated in rice flour. This low viscosity was also observed in jackfruit seeds’ starch when compared with starches from other sources such as native pinion starch and corn starch [5].

### 3.9. Prebiotic Effect of Jackfruit Seed Flour and Prebiotic Jackfruit Seed Extract

**Figure 4:**
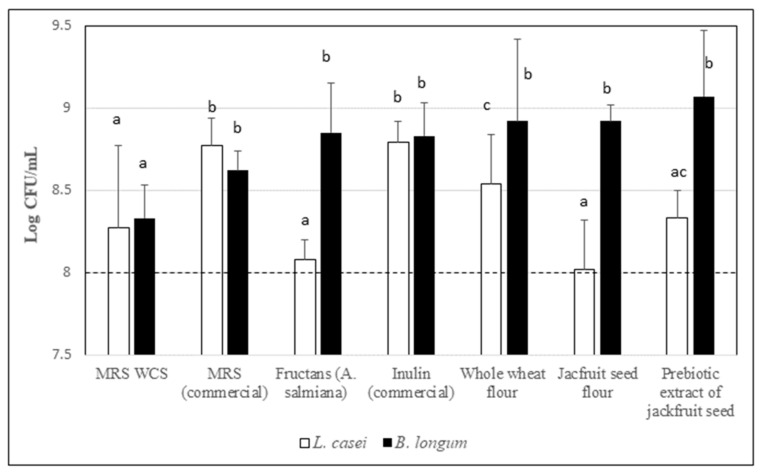
Prebiotic activity of jackfruit seed flour and prebiotic extract of jackfruit seed, compared with *A. salmiana* fructans, commercial inulin, whole wheat flour, commercial MRS and MRS without carbohydrates source (MRS-WCS). The values are the means ± S.E. Data with different letters are significantly different at *p* < 0.05.

One of the main characteristics of prebiotics is the stimulation of microorganism probiotics to improve the health of the gut microbiota [51]. For this reason, in this study the prebiotic activity of flour and prebiotic extract of jackfruit seed was evaluated by comparing it with other prebiotic ingredients. The results are shown in Figure 4 and are expressed as Log CFU/mL. For *L. casei*, significant (*p* < 0.05) differences were observed, commercial inulin and MRS (control positive) showed the highest bacterial growth: 8.79 ± 0.1 and 8.77 ± 0.1 Log CFU/mL, respectively. The flour from jackfruit seeds and *A. salmiana* fructans showed the lowest growth: 8.02 ± 0.1 and 8.08 ± 0.2, respectively. Jackfruit seed flour and *A. salmiana* fructans had a positive prebiotic score (higher than 8 Log CFU/mL), which was similar to that reported in other studies [21,52]. The prebiotic extract of jackfruit seed was significantly (*p* < 0.05) better fermented than the flour of jackfruit seeds by *L. casei*, which could be associated with the fact that in the prebiotic extract the non-digestible carbohydrates are better concentrated. *B. longum* showed a higher growth (9.07 ± 0.4 Log CFU/mL) in a prebiotic extract of jackfruit seed followed by jackfruit seed flour and whole wheat, where the value was the same: 8.92 ± 0.25 Log CFU/mL. No significant differences were observed, however, between them. Nevertheless, the prebiotic extract was significantly different (*p* < 0.05) with *A. salmiana* fructans, commercial inulin, the positive control (MRS COM) and the negative control (MRS WCS). The *A. salmiana* fructans were better (*p* < 0.05) fermented by *B. longum* than *L. casei*, and these results coincide with [16], where they evaluated the effect prebiotic of *A. salmiana* fructans on *Bifidobacterium* spp. and *Lactobacillus* spp. in vivo. For commercial inulin, however, the same (*p* > 0.05) behavior was observed in both bacteria. The growth of *B. longum* was higher in all tested prebiotics than the controls. The differences observed in bacterial growth can be associated with the ability that these bacteria have for fermenting non digestible carbohydrates. According to our results, we can say that *B. longum* is capable of degrading prebiotic compounds that are more complex than *L. casei* because jackfruit flour has a variety of nutrients like dietary fiber, polyphenols, resistant starch, and ketose. Additionally, it has been reported that *L. casei* preferentially degrades fructans with a low degree of polymerization [21]. These results suggest that jackfruit seed flour and the prebiotic extract from seeds induce the growth of probiotic bacteria equal to or better than other sources of prebiotics.

### 3.10. Anticancer Activity in Human Colon Epithelial Cells

**Figure 5:**
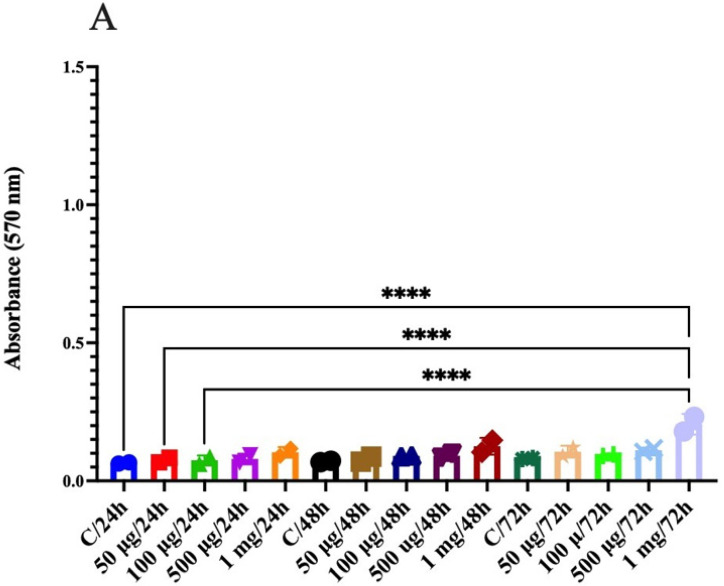

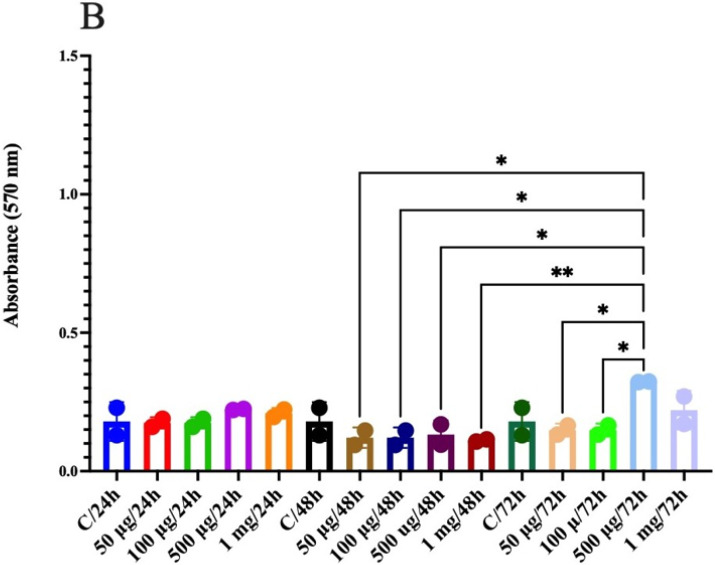

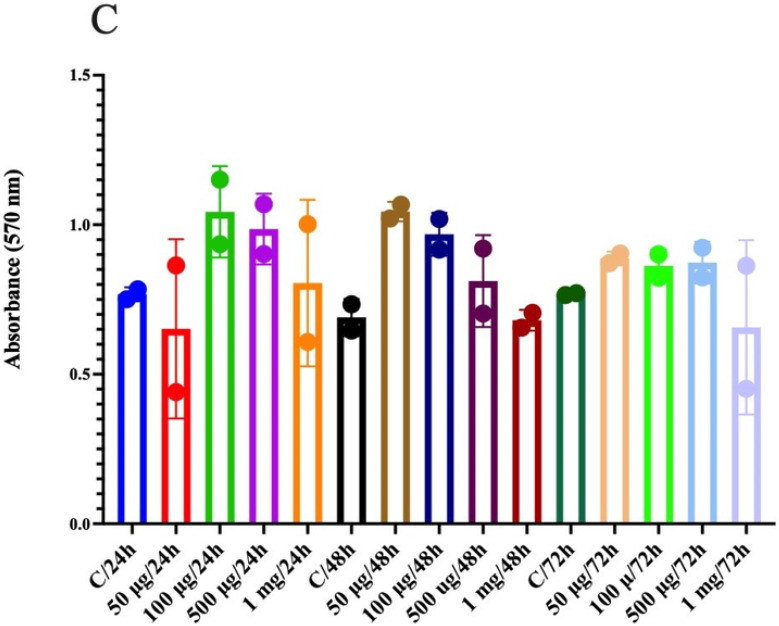
Cytotoxicity assay, was assessed with an MTT (thiazolyl blue tetrazolium bromide), at 24, 48 and 72 h. (**A**) CRL1831, (**B**) HT29, (**C**) SW480. (* *p* < 0.05, ** *p* < 0.01, **** *p* < 0.0001).

When natural compounds such as jackfruit flour are analyzed to evaluate cytotoxicity in normal colon cancer cells, it is expected to find that they have no cytotoxic effect on cellular stability. In the case of CRL1831 cells, all treatments maintained cellular balance. Statistically significant differences were only found when comparing the control (50 μg/mL and 100 μg/mL) against the 1 mg/mL concentration at 72 h, where a slight increase in absorbance can be observed, which means a greater amount of MTT (3-(4,5-dimethylthiazol-2-yl)-2,5-diphenyltetrazolium bromide) was transformed by the cell or greater cell viability (Figure 5A). When these same compounds were analyzed in colon cancer cell lines, however, as is the case of the HT29 cell line, we found that jackfruit flour has an anticancer effect at 50 μg/mL, 100 μg/mL, 500 μg/mL and 1 mg/mL at 48 h. Nevertheless, if 500 μg/mL is added and we wait 24 h more, the effect is the opposite (72 h), higher cell viability (Figure 5B). In the case of the SW480 cell line, there was no significant difference when comparing the groups because the advanced stage of this cancer, the cells cannot sense the extracts of the jackfruit flour. For this reason, they have an exacerbated viability. During biological processes, chemical species known as free radicals are generated, which are characterized by having an unpaired electron and by being very reactive. Of all the radicals, oxygen-derived reactive species (ROS) are of great interest because of the dual radical structure of these molecules and the large number of processes that generate them and in which they can be involved. The main ROS are superoxide anion (O_2_), hydroxyl radical (OH^+^), singlet oxygen, and hydrogen peroxide (H_2_O_2_). These radical species are involved in damage cell in such a way that oxidative stresses can lead to carcinogenesis, inflammatory diseases, cellular senescence, and neurodegenerative diseases, among other pathological processes. In the organism, there is an antioxidant protection system formed by enzymes and low molecular weight compounds. Two of the enzymes involved in the protection and consequently the maintenance of the oxidant/antioxidant balance are catalase (CAT) and superoxide dismutase (SOD).

**Figure 6:**
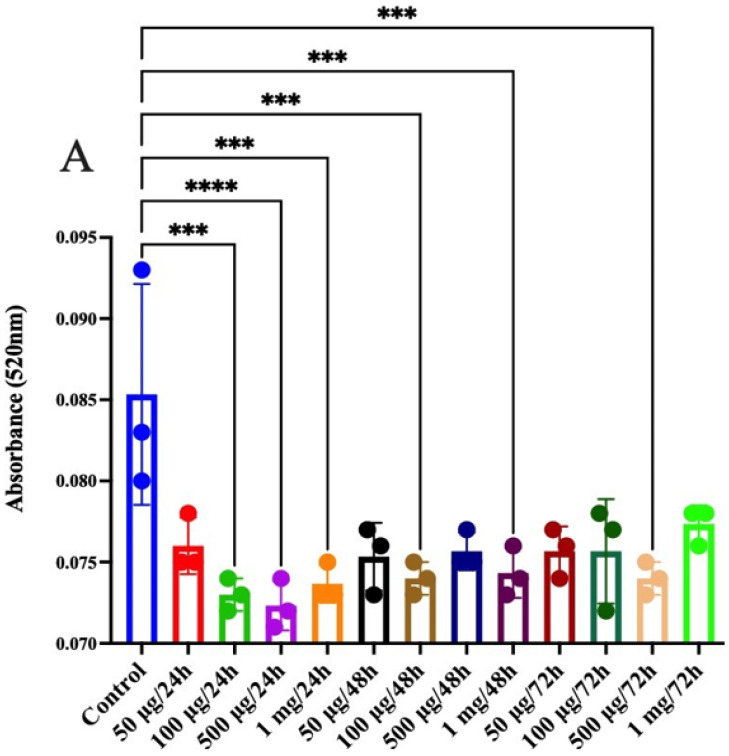

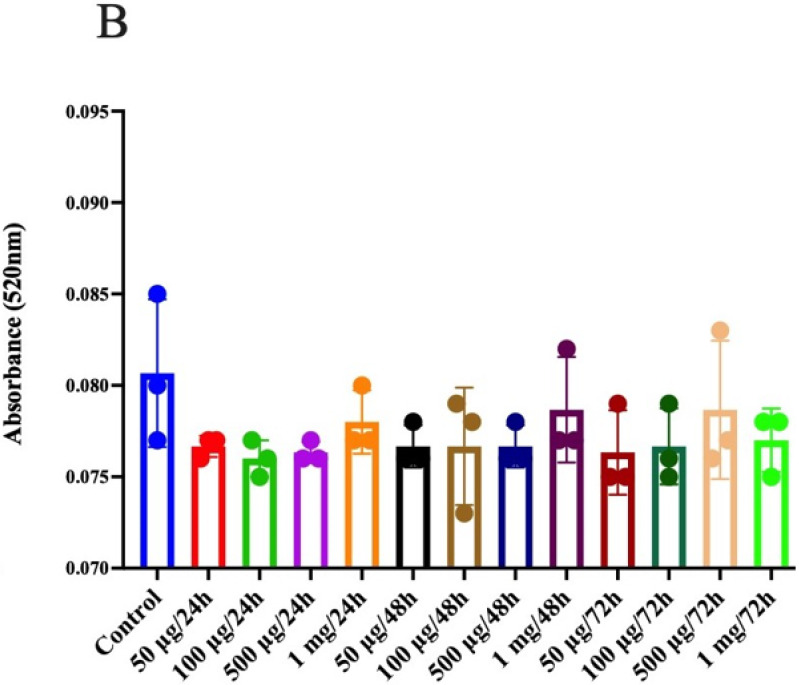

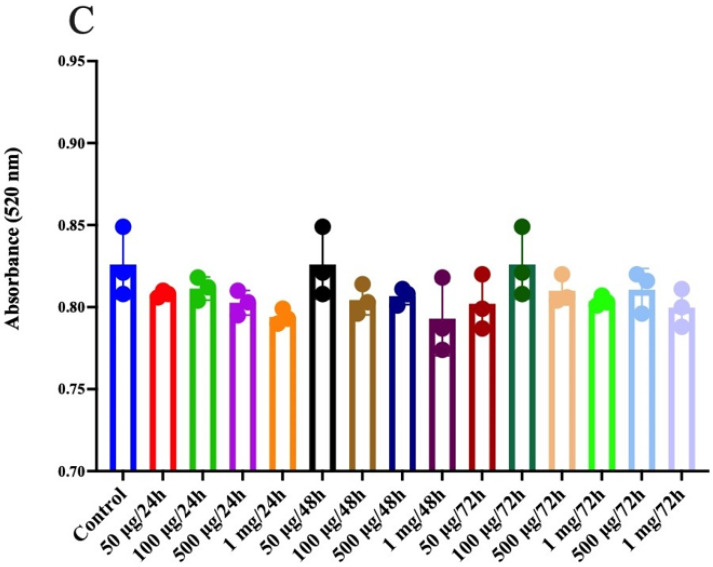
Catalase assay, (**A**) CRL1831, (**B**) HT29, (**C**) SW480. (*** *p* < 0.005, **** *p* < 0.0001).

Through the different treatments with jackfruit flour, it was observed that the control epithelial cells (CRL 1831 from a normal colon) presented a significant difference when 100 μg/mL, 500 μg/mL, and 1 mg/mL of jackfruit flour was added at 24 h, as well as 100 ·g/mL and 1 mg/mL of the jackfruit flour at 48 h, and finally 500 μg/mL of proteins of jackfruit flour at 72 h (Figure 6A). No significant differences were found, however, for HT29 (Figure 6B) and SW480 (Figure 6C) cells; both lines are colon cancer but at a different stage: stage 1–2 and stage 3–4, respectively. CAT levels in SW480 cells are tenfold higher than even in normal and HT29 cells because of the excessive stress that stage 3–4 cells are under. 

**Figure 7:**
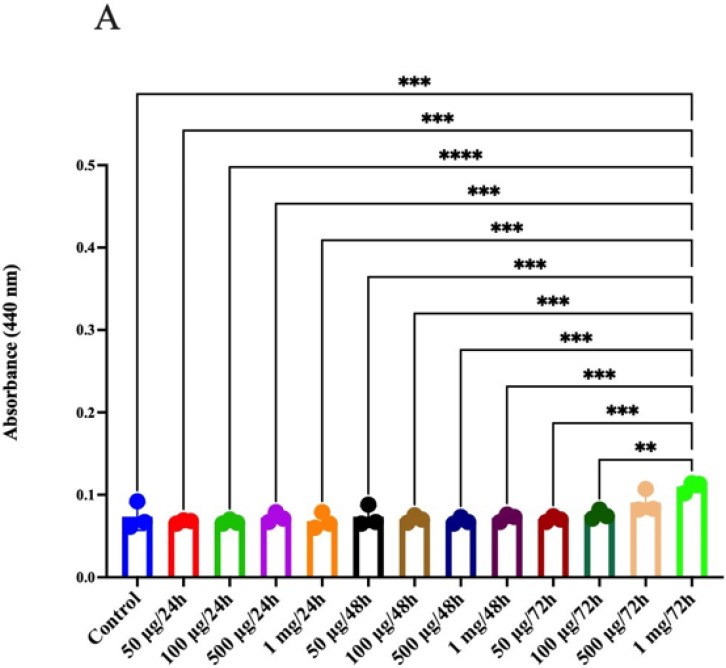

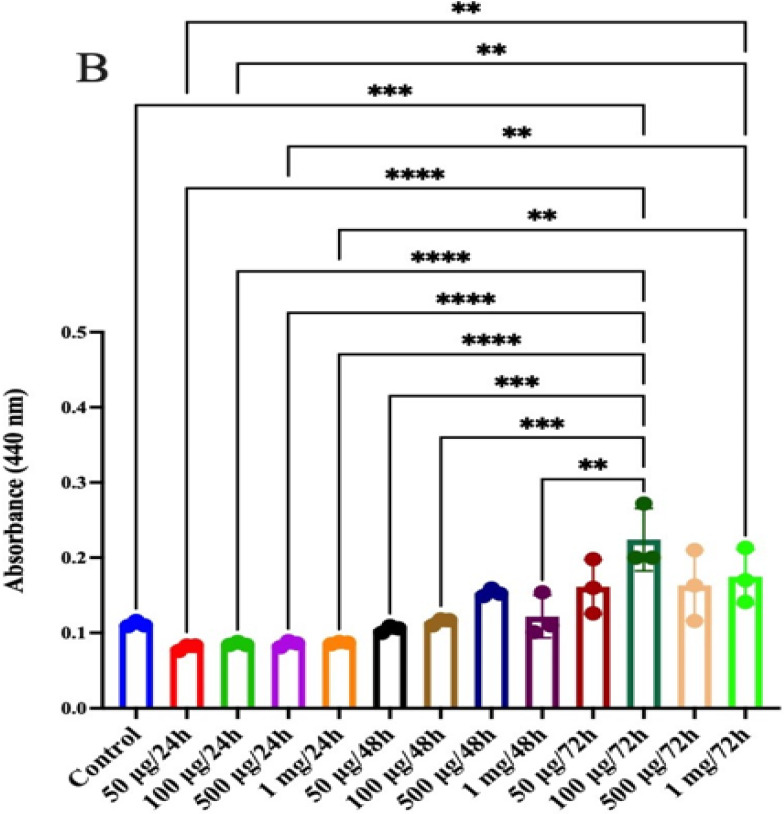

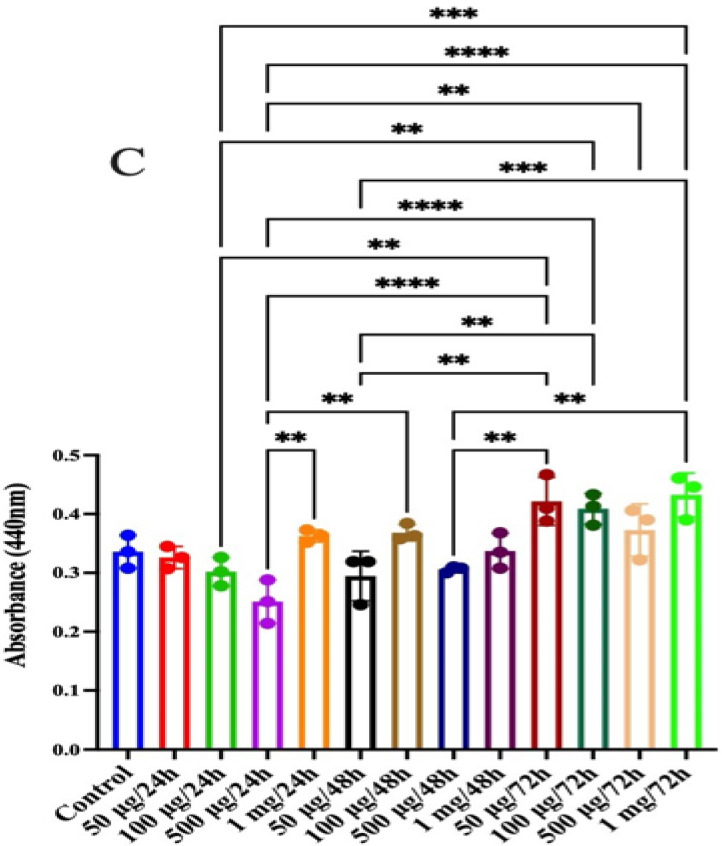
SOD assay. (**A**) CRL1831, (**B**) HT29, (**C**) SW480. (** *p* < 0.01, *** *p* < 0.005, **** *p* < 0.0001).

For the CRL 1831 cells, in the case of SOD, it was found that the highest concentration of jackfruit flour (1 mg/mL) at the maximum of 72 h showed significant differences when compared with the control and even with each treatment (Figure 7A). In the case of the HT29 cell line, significant differences were found at 100 μg/mL at 72 h, when compared with the other treatments and even with the control. Similarly, for the concentration of 1 mg/mL at 72 h, differences were found with the four other treatments. (Figure 7B). In the case of the SW 480 cell line, significant differences were found when comparing 1 mg/mL at 72 h with the four treatments: 50 μg/mL and 500 μg/mL at 48 h, and 100 μg/mL and 1 mg/mL at 24 h. For that, we consider that the best time to see the effect is at 72 h using 1 mg/mL of concentration (Figure 7C). If we compare only the controls for SOD, we can see an increase in the SW480 cell line for stage 3–4. Therefore, these colon cancer tumor cells are under great oxidative stress, which can generate continuous genetic changes that are manifested as an increase in chromosomal abnormalities and mutations and in turn can lead to tumor genesis and spreading (metastasis). Colon cancer is one of the most prevalent malignant diseases and a major health problem throughout the world, causing a high rate of mortality [53]. Strategies to prevent colon cancer consisting of improved diet components have become an important tool and need much more understanding for its proper use. The main finding of this work is that jackfruit flour shows antioxidant and antiproliferative effects on a colonic cancer cell line. These effects depend on the stage of cancer progression and were not observed in more advanced stages; they were also milder in the control epithelial cell line, which shows the potential for jackfruit flour as a preventive nutraceutical.

Tumorigenesis, as the hallmark of cancer, consists of a dysregulation of cellular proliferation, leading to uncontrolled cell growth. Therefore in this work we tested the effect of jackfruit flour on cell viability in a control colonic epithelial cell line and in two colonic cancer cell lines. Jackfruit flour did not alter cell viability in a controlled colonic epithelial cell line, either by showing cytotoxicity nor by inducing proliferation. It has been reported that artocarpin, a phenolic compound found in the wood of jackfruit decreased cell viability in five different colon adenocarcinoma cell lines (DLD1, HCT15, HCT116, HT29, and SW480) but not in normal colon fibroblast cells (CCD-18Co). It induced apoptosis and autophagy, inducing G1 phase cell cycle arrest by targeting Akt1 and Akt 2 kinase activity, and reduced tumorigenesis in vivo [54].

On the other hand, the flour decreased cell viability in the HT29 cell line, which is a cancerous cell line, and thus showed anticancer effects. Reference [55] recently reported that water-soluble polysaccharides obtained from jackfruit did not affect viability in HT29 nor SW620 colonic cancer cell lines after 24 or 48 h as tested by a neutral red assay. Nevertheless, an MTT assay of these same cell lines showed a gradual decrease in cell viability with increasing concentrations of polysaccharide extracts. The anti-proliferative effect was also supported by a high antioxidant activity of these polysaccharide extracts.

Viability in the SW480 cell line was very high in basal conditions, which is expected since it is a cell line from advanced cancer stages characterized by Broders’ classification as grade 4, and shows multiple mitosis and high ROS production [56]. Jackfruit flour did not alter viability in these cells at any concentration or incubation time. A possible explanation for this is that this cell line shows a very high basal proliferation rate related to mutations and a loss of apoptosis, which has been well characterized in this cell line [57]. A very interesting feature of the jackfruit flour treatment was its antioxidant activity in colonic cells. Oxidative stress consists mainly of the excessive formation of oxygen-containing chemical species, such as superoxide, hydroxyl and hydrogen peroxide by enzymatic and non-enzymatic cellular pathways. Oxidative stress in a healthy background is naturally counterbalanced by specific enzymes (catalase, SOD, etc.) that transform ROS into harmless chemical species, such as oxygen and water. This process is deeply involved in the initiation and progression of colorectal cancer [58], given that the colon is chronically exposed to oxidizing compounds, toxins and iron. An oxidative environment promotes chromosome damage and thus mutations that lead to increased proliferation and tumour formation [59]. It is not surprising then that antioxidant strategies have been widely proposed as a strong preventive tool against cancer. In this work we reported many compounds found in jackfruit extracts such as polysaccharides and polyphenols which have antioxidants activity. In this work we measured catalase and SOD activity as a means of evaluating the effect of jackfruit flour on oxidative stress response in colonic cell lines. We observed that control epithelial cells showed basal catalase activity, which significantly decreased after incubation with jackfruit flour at different doses after 24, 48 and 72 h. Nevertheless, SOD activity did not decrease, which might mean that catalase activity is sufficient in controlling ROS formation. Regarding cancerous cells, in HT-29 cells there was a slight decrease in catalase activity, which was not significant, whereas SOD activity decreased at 24 h of incubation but increased over longer periods of time. This might be explained by increased oxidative stress because of the incubation in media for longer periods of time. Regarding SW480 cells, catalase and SOD activities were high under all conditions. These high levels of ROS have been previously reported for this cell line [60], which is highly proliferative and thus very active metabolically, leading to a high production of ROS that could not be scavenged efficiently by jackfruit flour.

## 4. Conclusions

Drying curves of jackfruit seeds were satisfactorily described by mathematical models based on Page’s equation and can be used to calculate the required or desired moisture content of samples at 50, 60 and 70 °C. Jackfruit seeds flour obtained at 60 °C is a nutritional food because of the presence of proteins, monosaccharides, oligosaccharides and polysaccharides, as well as omega-3 and omega-6 fatty acids. This flour also provides bioactive ingredients, such as total dietary fiber, polyphenols and antioxidant properties. Additionally, this flour exhibits good functional properties, such as water and fat absorption capacity, swelling powder and emulsifier capacity. The flour and prebiotic extract of jackfruits seed showed a potential prebiotic effect compared with other prebiotic sources. Moreover, jackfruit flour has a protective and preventive effect in colon cancer cells. Additional research, however, is needed to optimize the application of this flour, to assess its effect as a texture adjuvant in food matrices like meat products, bread, beverages or to develop new food products with functional ingredients that may prevent intestinal diseases.

## Figures and Tables

**Table 1:** Coefficients obtained from the Page model according to experimental data evaluated at different temperatures.

Model	50 °C		60 °C		70 °C	
Page	Coefficients	r^2^	Coefficients	r^2^	Coefficients	r^2^
	k = 0.0129	0.999	k = 0.0402	0.996	k = 0.0248	0.999
	*n* = 0.9618		*n* = 0.7956		*n* = 0.9593	

**Table 2:** Proximate composition of jackfruit seed flour in dry basis.

Component g/100 g	Means ± Standard Deviation
Moisture	6.54 ± 0.03
Ash	4.19 ± 0.13
Total carbohydrates	73.87 ± 1.06
Reducing sugars	6.11 ± 0.04
Total dietary fiber	31.59 ± 11.14
Proteins **	14.07 ± 0.15
Lipids	1.3 ± 0.24

** Quantified by the Kjeldahl method. Conversion factor of *n* = 6.25.

**Table 3:** Carbohydrate composition of jackfruit seed flour.

Carbohydrate	Content (mg/g)
Compounds greater than 5 degrees of polymerization	20.0 ± 1.3
1-Kestose	3.8 ± 1.43
Sucrose	35.5 ± 5.4
Glucose	29.4 ± 2.7
Fructose	22.9 ± 6.2

**Table 4:** Fatty acid composition of jackfruit seed flour.

Fatty Acid	Content (g/100 g)
Palmitic acid (C16:0)	36.20 ± 2.68
Stearic acid (18:0)	3.54 ± 0.50
Tricosanoate acid (C23:0)	9.39 ± 0.51
Oleic acid (C18:1)	4.15 ± 1.38
Linoleic acid (C18:2)	35.11 ± 0.59
Linolenic acid (C18:3)	2.82 ± 0.25
Arachidonic acid (C20:4)	3.82 ± 0.23
Eicosapentaenoic acid (20:5 (n-3))	4.01 ± 0.65
Docosadienoic acid (C22:2 (n-6))	0.97 ± 0.02
Total saturated fatty acids	49.13 ± 4.90
Total monounsaturated fatty acids	4.15 ± 2.30
Total polyunsaturated fatty acids	46.72 ± 2.12
**ω**6/**ω**3	5:1

**Table 5:** Polyphenol content and antioxidant activity of jackfruit seed flour at the three evaluated temperatures.

Temperature (°C)	Polyphenol Content (mg of Gallic Acid/g of Sample)	DPPH Radical (µmol Trolox/100 g)	FRAP (µmol Trolox/100 g)
50	2.65 ± 0.86 ^a^	1579.51 ± 1.91 ^a^	1717.20 ± 3.9 ^a^
60	2.42 ± 0.74 ^a^	1607.87 ± 2.14 ^b^	901.45 ± 5.84 ^b^
70	2.39 ± 0.92 ^a^	1617.48 ± 0.72 ^b^	489.77 ± 2.87 ^c^
Fisher	0.539	0.0211	<0.0001

Means within a column followed by different letters are significantly different (*p* < 0.05).

**Table 6:** Water solubility and swelling power of jackfruit seed flour evaluated at different temperatures.

Temperature (°C)	Water Solubility (%)	Swelling Power (g/g)
25	0.087 ± 0.001 ^a^	4.062 ± 0.010 ^a^
40	0.1586 ± 0.0001 ^a^	4.779 ± 0.012 ^a^
50	0.1527 ± 0.007 ^a^	5.022 ± 0.045 ^a^
60	0.1580 ± 0.001 ^a^	5.163 ± 0.041 ^a^
70	0.1492 ± 0.004 ^a^	5.515 ± 0.088 ^a^
Fisher	0.069	0.112

Means within a column followed by different letters are significantly different (*p* < 0.05).

## Data Availability

Data used to support the findings of this study are available from the corresponding author upon request.
